# Notes and News

**Published:** 1898-10-29

**Authors:** 


### NOTES AND NEWS.

(Collected and Communicated.)

At the annual meeting of the Sheffield Infirmary an anonymous gift of £20,000 was announced.

Conscientious objectors apparently abound in Keighley. Eight hundred exemptions were granted in one day, and altogether exemptions affecting 3,700 children have been granted.

The Local Government Board inquiry as to the excess of expenditure beyond the estimated cost of the Brook Fever Hospital is concluded. Mr. W. E. Knollys, the inspector, stated at the last sitting that the Metropolitan Asylums Board appeared to have left expenditure unreservedly in the architect's hands. It was pointed out, nevertheless, that very little ordered by the architect could be regarded as unnecessary, and also that the preparation of the estimates was greatly hurried.

It is urged by the friends of the Royal Ear Hospital that the accommodation now available is inadequate, and a plea for extension is being put forward. The out-patients are numbered at 2,500 during the year, or rather less than an average of seven per diem; the beds at present available are nine. Even when the accommodation asked for is secured, only 15 appear to be provided, so that the new hospital will, as far as in-patients are concerned, equal about half the size of one ward at one of the larger hospitals. The outlay is estimated at £13,200, or about £880 per bed.

The methods adopted at the Royal Infirmary, Edinburgh, for the election of members of the medical staff of the institution have recently been questioned. The salaried posts are subject to annual election by the board of management, seven of which at present are medical men. It is stated that for some time past the holders of the two pathological posts have been specially regarded as favoured candidates for appointments in the clinical medicine ranks, and this is the point to which objection is raised. It is contended that to perform the enormous amount of work now entailed upon the pathologists nearly all their time is required. On these and other grounds the favour shown to holders of pathological posts at the infirmary is deprecated.

The Stockport Town Council intend inviting the Lord Mayor-Elect to lay the foundation-stone of the enlargements to the infirmary.

Moretonhampstead is to have a cottage hospital. It is to be erected on a site given by the Hon. W. F. Smith and Lady Esther Smith, and will be endowed by Mr. G. Wills, of Poppedon, in memory of his wife. The initiation of the scheme is due to Mr. Wills.

The Duke and Duchess of Westminster opened a hospital at Winsford in the salt manufacturing district of Cheshire. The hospital, which is to bear the name of the Albert Infirmary, is the gift of Mr. Verdin, the building having previously been his private residence. Up to now the nearest hospital to which accident cases from Winsford and Middlewich could be taken was the Victoria Infirmary at Northwich, also a gift from the same family.

The proceedings at a recent meeting of the Bradford Board of Guardians show a progressive spirit. The minutes of the Infirmary Committee stated that a deputation had visited Birmingham to study the system which prevailed there with regard to medical attendance at the Union hospitals and workhouse. After consideration, it was resolved by the committee: "That it be a recommendation to the Board that a scheme be adopted for the appointment of two visiting medical officers and two resident medical officers, with a salary of £100 per annum each, for this workhouse and hospitals; that the Local Government Board be requested to sanction this arrangement, and informed that it is the intention of the Guardians to take into consideration the providing of separate administration for the hospitals, and that arrangements are in contemplation for the obtaining of the consent of the Local Government Board to the erection of a new female hospital and administrative block."

In an address recently given before the Sanitary Institute, Mr. Garrett Horder, the honorary secretary of the Hospital Reform Association, urges that the aggregation of large numbers of sick people in the out-patient department of our hospitals and infirmaries is unnecessary, is detrimental to the patients, and is likely to cause the spread of infectious disorders. He urges, also, the exclusion of trivial cases, a thing that has always been urged by everyone who has seriously tackled the question, if only it could be done; but he has to confess that "no one but a skilled medical man can be trusted to say whether a case is serious or trivial." For this reason he thinks it desirable that all large hospitals should have a special resident medical officer, whose duty it should be to weed out infectious cases from the out-patient department, and to discriminate between trivial cases and such as really require hospital treatment—a system, however, which is already in operation at some of our hospitals. Finally, he wishes to see provision made for the periodical inspection of all hospitals by the medical officer of health of the district, and for the publication by the sanitary authority of a report dealing with any defects which may come under his notice. We see no objection to any of these proposals. We think, however, that we have heard of them before.

